# A bifid gallbladder? A challenging laparoscopic cholecystectomy

**DOI:** 10.1016/j.ijscr.2024.109760

**Published:** 2024-05-17

**Authors:** Sleiman Marwan-Julien, Jelip Annamaria, Toso Christian, Delaune Vaihere

**Affiliations:** a Division of Visceral Surgery, Department of Surgery, Geneva University Hospitals, Geneva, Switzerland; b Laboratory of Transplantation and Hepatology, University of Geneva, Geneva, Switzerland

**Keywords:** Surgery, Gallbladder, Cholecystitis, Challenging cholecystectomy, Cystohepatic triangle, Critical view of safety

## Abstract

**Introduction:**

The modern-day gold standard treatment of acute cholecystitis is laparoscopic surgery. It is, however, associated with a higher risk of bile duct injury (0.1 %–1.5 %) when compared to the open approach.

**Case presentation:**

We report a case of a patient with an acute cholecystitis in which we performed a laparoscopic cholecystectomy. We faced a destabilizing anatomy with what looked like the gallbladder and an unidentified mass, interpreted as a possible common bile duct cyst. Careful dissection allowed us to determine that what looked like a common bile duct cyst was a dilatation of “Hartmann's pouch” due to a large gallstone.

**Discussion:**

Laparoscopic cholecystectomy reduces length of hospitalization and enhance intra-operative and postoperative morbidity compared with open cholecystectomy. It may increase the risk of bile duct injury, notably in an acute setting due to inflammation and an unclear anatomy. Hartmann's pouch with the infundibulum can sometimes unexpectedly be present beneath the common hepatic duct. In order to avoid bile duct injury, notably in an acute setting, a surgical technique was developed, the Critical View of Safety. It is a method whose sole aim is to secure identification of the cystic structures.

**Conclusion:**

Understanding the anatomy allowed for an ultimately safe laparoscopic cholecystectomy. It is strongly advised that, in the event of atypical anatomy, a second opinion is asked of another and/or more experimented surgeon.

## Highlights

• Laparoscopic approach for cholecystectomy is now considered the gold standard. It is also the most frequent cause of bile duct injury. This is most probably multifactorial, but the risk tends to increase when the operation is difficult as a result of inflammation, leading to a difficulty in the identification of anatomical structures
• In order to avoid bile duct injury, notably in an acute setting, a surgical technique was developed, the Critical View of Safety (CVS). It is a method whose sole aim is to secure identification of the cystic structures
• It is advisable that operating surgeon should rest and ask for a second opinion from another surgeon in the situation of any unexpected finding, a difficult gallbladder, unusual anatomy, or a difficult dissection

## 1 Introduction

Acute cholecystitis is most often due to the obstruction of the cystic duct, usually by a gallstone, followed by distension and subsequent inflammation of the gallbladder. Twenty percent of patients hospitalized for a biliary pathology, present a diagnosis of acute cholecystitis [1]. Around 95 % of patients with acute cholecystitis have calculous cholecystitis, only 5 % have an acalculous cholecystitis [2].

Acute cholecystitis usually induces unremitting right upper quadrant pain, nausea, vomiting, and sometimes fever. Severe acute cholecystitis may evolve to gangrenous cholecystitis, with necrosis of the gallbladder wall. Surgical removal of the gallbladder remains the only definitive treatment for acute cholecystitis [3].

The laparoscopic approach for cholecystectomy is now considered the gold standard. It is also, however, the most frequent cause of bile duct injury. This is most probably multifactorial, but the risk tends to increase when the operation is difficult as a result of inflammation, leading to a difficulty in the identification of anatomical structures [3]. The presence of a Hartmann's pouch, a small outpouching that can be seen at the infundibulum of the gallbladder, may also increase the difficulty in recognizing anatomical structures. It is a rare congenital anomaly of the gallbladder [4] and may be a site of gallstone impaction. The size of the pouch can be variable and may obscure the cystic duct and the cystohepatic, or Calot's, triangle [5].

Timing of the cholecystectomy for acute cholecystitis has been widely debated due to an increased risk of lesions in the acute setting. However, current literature, supported by a 2013 Cochrane review, now supports early cholecystectomy for acute cholecystitis [6]. They compared 488 patients who underwent cholecystectomy in less than 7 days vs. more than 6 weeks after onset of symptoms. They observed no difference in rates of conversion-to-open (early 19.7 vs. delayed 22.1 %), postoperative complications (early 6.5 vs. delayed 5.0 %) or the incidence of bile duct injuries (early 0.4 vs. delayed 0.9 %) between the groups. In 2015, Coccolini et al. [7] also showed that post-operative morbidity, mortality and hospital stay are reduced when performing laparoscopic cholecystectomy in the acute setting.

We report a case of an acute cholecystitis, operated on in an acute setting, with an anatomical conundrum, leading to a challenging laparoscopic cholecystectomy. This work has been reported in line with the SCARE criteria [8].

## 2 Case presentation

A male, 50 year-old patient, with no particular medical or surgical history, with no history of biliary colic or known gallstones, presented in the emergency department with a right upper-quadrant abdominal pain since 2 days. He complained of nausea, but no vomiting; there was no change in urine or stool colour. He has normal vital signs (68 bpm, 152/87 mmHg blood pressure), and no fever.

Upon clinical examination, patient was not icteric. He has pain at the palpation of the right upper quadrant with a positive Murphy sign.

**Fig. 1:**
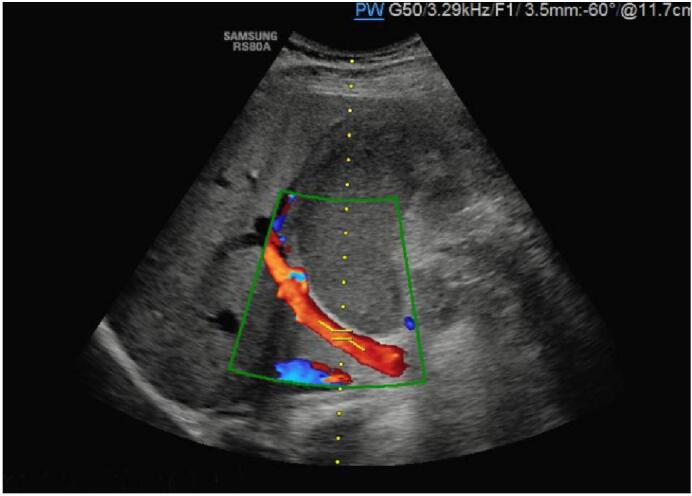
Abdominal US showing no anatomical abnormalities on the gallbladder.

Laboratory tests showed systemic inflammation with a white blood cell count of 15 G/l, and CRP 100 mg/l. Liver enzymes were slightly elevated: GGT 203 U/l, total bilirubin 32 μmol/l, conjugated bilirubin 12.8 μmol/l). Blood lipase was not tested in the emergency department. An abdominal ultrasound confirmed the diagnosis of acute cholecystitis with the presence of a gallstone in the infundibulum with, no unusual anatomy was reported by the radiologist. (Fig. 1).

Although liver enzymes were slightly elevated, our local algorithm dictates immediate laparoscopic cholecystectomy [9].

**Fig. 2:**
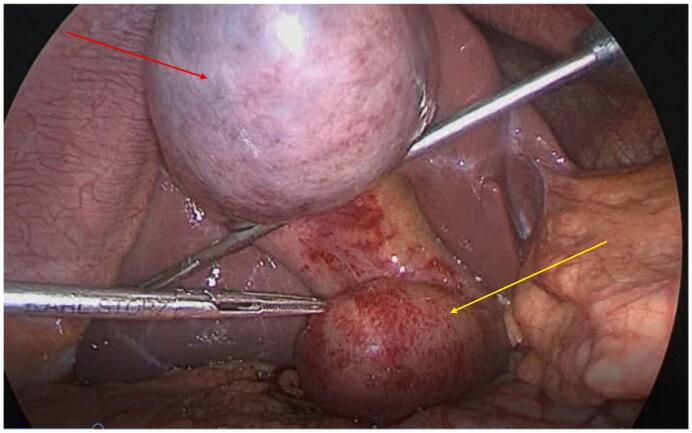
Acute cholecystitis with: yellow arrow –unidentified pouch Red arrow -gallbladder. (For interpretation of the references to colour in this figure legend, the reader is referred to the web version of this article.)

In our institution, gallbladder surgery is performed daily by senior registrars specialized in general surgery. Laparoscopic cholecystectomy was performed the day after admission, a Saturday morning, by the on-call surgical team (resident and senior registrar). We were surprised by an unusual anatomy, resembling a bifid gallbladder, with an unidentified bulging pouch in the region of the cystohepatic triangle (Fig. 2). Our initial suspicion was that of a large cyst of the common bile duct. We mobilized our on-call consultant who was, luckily, an hepato-bilio-pancreatic surgeon, who was also unsure of the anatomical structures observed. After careful laparoscopic dissection, we discovered this mass to be intimately attached to the gallbladder. However, we still hadn't identified this structure. We decided to puncture and empty the gallbladder, in order to release its tension and to see if the second pouch would also deflate. This second pouch deflated as well, hinting that it belonged to the gallbladder.

**Fig. 3:**
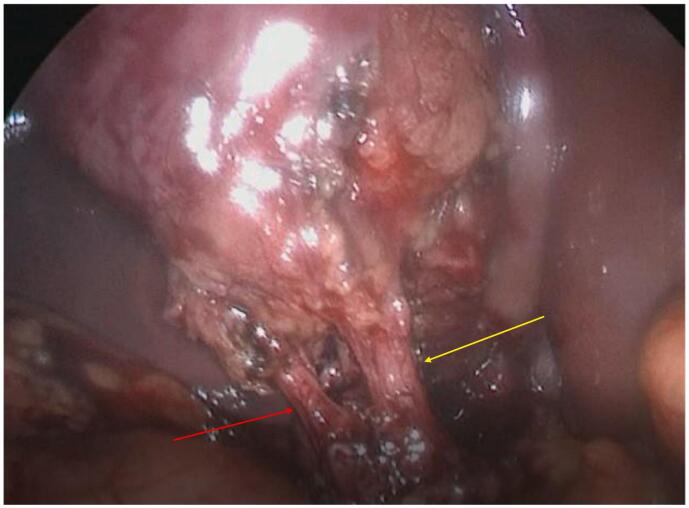
Critical view of safety, having dissected Calot's triangle and identified only 2 structures entering the gallbladder Red arrow: Cystic artery Yellow arrow: Cystic duct. (For interpretation of the references to colour in this figure legend, the reader is referred to the web version of this article.)

We very carefully started the dissection of the cystohepatic triangle under the second pouch. This finally allowed us to mobilize the content of this pouch, which turned out to be a very large gallstone in Hartmann's pouch. We then clearly identified the critical view of safety with only 2 structures entering in this pouch of the gallbladder, the artery and the cystic duct (Fig. 3).

**Fig. 4:**
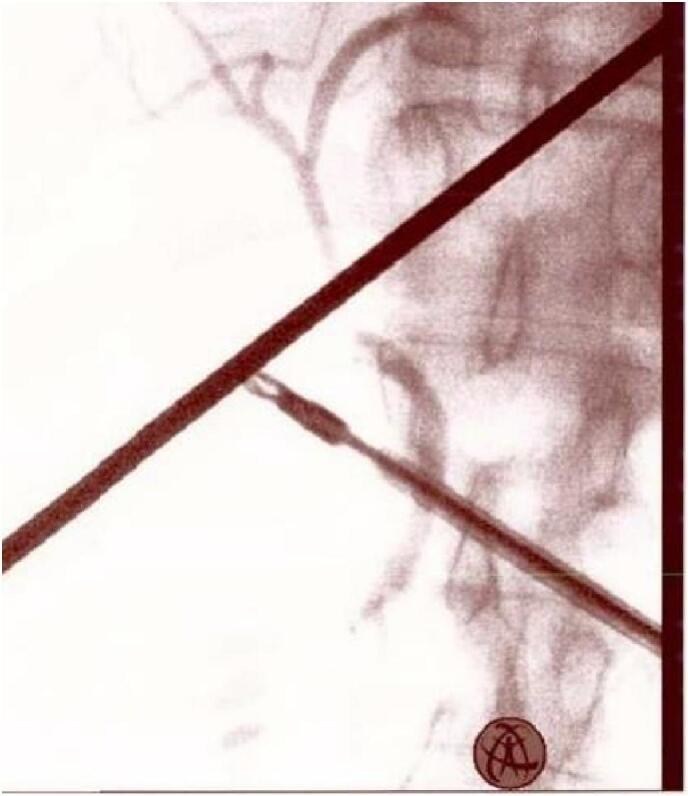
Intraoperative Cholangiography showing no abnormal biliary anatomy.

An intraoperative cholangiogram confirmed the anatomy and the vacuity of the common bile duct (Fig. 4). The intervention was safely completed laparoscopically. The patient was discharged the next day.

## 3 Discussion

Laparoscopic cholecystectomy reduces length of hospitalization intra-operative and postoperative morbidity compared to open cholecystectomy [1]. It may, however, increase the risk of bile duct injury, notably in an acute setting, due to inflammation and an unclear anatomy [1]. Laparoscopic cholecystectomy is associated with an overall complication rate of approximately 10 % with a higher risk of biliary injury (0.1 %–1.5 %) when compared to the open cholecystectomy (0.1 %–0.2 %) [10]. Factors predisposing to bile duct or vasculo-biliary injuries are related to anatomy, structural misidentification, disease-related pathology, and improper technique [10]. The most common mechanism of such injuries involves the misidentification of the common bile duct or common hepatic duct as the cystic duct, or the misidentification of the hepatic artery as the cystic artery. A Hartmann's pouch within the infundibulum can sometimes unexpectedly be present beneath the common hepatic duct. This can mislead the surgeon into assuming that the common bile duct or the common hepatic duct is the cystic duct [11]. Findings such as these become a difficulty to the surgeon and a risk to the patient [12].

The major causes of bile duct injury have been summarized to three factors:

(1) Technique – Related to surgeon's experience and performance;
(2) Pathology – The extent of hilar inflammation;
(3) Anatomy – The presence of anomalies of the bile duct. [13]

In order to avoid bile duct injury, notably in an acute setting, a surgical technique was developed, the Critical View of Safety (CVS). It is a method whose sole aim is to secure identification of the cystic structures [14]. Today, CVS is widely taught and used [15]. To achieve CVS, three requirements must be met [16]. First, the cystohepatic triangle must be dissected from fat and fibrous tissue. Second, the lowest part of the gallbladder must be separated from the cystic plate. The third requirement is that only two structures should be seen entering the gallbladder.

When these three conditions are met, CVS has been attained and cholecystectomy can be performed. It does not require that the common bile duct be exposed.

In our institution, routine intraoperative cholangiogram is the norm. However, in those institutions where it is not, and/or whenever any doubts persist on the anatomy, it is highly recommended to perform an intraoperative cholangiogram (IOC) as an assessment of the biliary anatomy. It allows for the possible prevention of bile duct injury; in case of a bile duct injury, it identifies and judges the extent of the injury, thus permitting the planning of a repair strategy. Several large retrospective data sets report association of IOC with lower rates of bile duct injuries [17].

A recent meta-analysis confirmed the importance of obtaining the CVS described by Strasberg as a significant protective factor in the prevention of bile duct injury and/or hemorrhagic complications [18]. The presented case, which may be encountered during a surgeon's career, illustrates the critical need for performing CVS when faced with an unclear anatomy, even more so in an emergency setting.

Atypical anatomical findings need to be quickly identified and warrant the correct management at the time of surgery to avoid complications. Clear identification of the anatomy is mandatory.

Conversion to an open procedure is another option. A word of caution, conversion does not protect against bile duct/vascular injury [19].

There are other bailout strategies for a difficult gallbladder like aborting the procedure altogether and referring to a specialist, gallbladder drainage in the acute setting (tube cholecystostomy), subtotal cholecystectomy and fundus first cholecystectomy [20,21].

It is advisable that the operating surgeon should rest and ask for a second opinion from an hepato-bilio-pancreatic surgeon in the situation of any unexpected finding, a difficult gallbladder, unusual anatomy, or a difficult dissection.

## 4 Conclusion

Laparoscopic cholecystectomy can be challenging in the setting of acute cholecystitis but is recommended as a standard of care in the literature. Our presented case illustrates the importance of obtaining the Critical View of Safety, notably in emergency surgery, in order to decrease the risk of bile duct or vasculo-biliary injury. It is strongly advised that, in the event of atypical anatomy, a second opinion is asked of another and/or more experimented surgeon.

## Ethical approval

No ethical approval was necessary from our institution.

## Funding

There is no funding for this work.

## CRediT authorship contribution statement

Sleiman Marwan-Julien: Data curation, Writing original draft, Writing & Review

Jelip Annamaria: Data curation, Writing original draft, Writing & Review

Toso Christian: Supervision and Review

Delaune Vaihere: Writing and Review

## Guarantor

Sleiman Marwan-Julien.

## Content

Written informed consent was obtained from the patient.

## Declaration of competing interest

All authors declare no conflicts of interest.
